# Effect of Age and Sex on Normalized Automated DECT-Derived Pulmonary Iodine Concentration

**DOI:** 10.3390/diagnostics16081134

**Published:** 2026-04-10

**Authors:** Thomas Schömig, Andrii Sabov, David Zopfs, Nedim Christoph Beste, Florian J. Fintelmann, Alexander Christian Bunck, David Maintz, Roman Johannes Gertz, Nils Große Hokamp

**Affiliations:** 1 Institute for Diagnostic and Interventional Radiology, Faculty of Medicine and University Hospital Cologne, University of Cologne, Kerpener Straße 62, 50937 Cologne, Germanydavid.zopfs@uk-koeln.de (D.Z.); nedim.beste@uk-koeln.de (N.C.B.); alexander.bunck@uk-koeln.de (A.C.B.); david.maintz@uk-koeln.de (D.M.); roman.gertz@uk-koeln.de (R.J.G.); nils.grosse-hokamp@uk-koeln.de (N.G.H.); 2 Department of Radiology, Division of Thoracic Imaging and Intervention, Massachusetts General Hospital, Boston, MA 02114, USA; fintelmann@mgh.harvard.edu

**Keywords:** dual-energy computed tomography, iodine map, pulmonary perfusion, pulmonary embolism

## Abstract

**Background/Objectives:** Dual-energy CT (DECT) enables iodine quantification as a snapshot perfusion indicator. Understanding pulmonary iodine distribution in lung-healthy individuals is crucial for clinical applications. This study aimed to automate iodine quantification and assess demographic effects in a lung-healthy reference cohort. **Methods:** This retrospective cohort study included 112 adults (53% female, mean age 60.3 ± 16.6 years) who underwent repeated portal venous phase chest DECT on a spectral detector dual-layer scanner between 2016 and 2019 at an academic medical center. Patients had dermato-oncological diseases but no visible thoracic tumors. Automatic lung volumetry was merged with reconstructed iodine maps to assess volume and mean iodine concentrations of each lung lobe. Pulmonary iodine perfusion ratios (PIPRs) were calculated by normalizing the pulmonary iodine density against iodine concentration in the portal vein and the main pulmonary artery (mPA). **Results:** Mean lung volume (f: 3.9 L vs. m: 5.2 L) and iodine concentration (f: 0.87 mg/mL vs. m: 0.69 mg/mL) differed between ages. However, no difference was observed when comparing PIPRs after normalizing against the iodine level in the mPA. PIPR_mPA_ were consistent across two timepoints (r = 0.88) and decreased with increasing age (≤50 years: 0.18 vs. ≥70 years: 0.15). **Conclusions:** This study demonstrates that automated pulmonary iodine quantification is feasible. Normalized pulmonary iodine concentration is a more reliable and effective method for evaluating iodine distribution. Our study also highlights the need to account for sex and age variations in future research and clinical applications.

## 1. Introduction

In recent years, advancements in dual-energy computed tomography (DECT) have enhanced tissue characterization through material decomposition. The distinct, energy-dependent absorption characteristics of iodine in DECT enable the generation of quantitative iodine maps (IMs) [1]. By visualizing the absolute iodine concentration in mg/mL across various organs, these maps offer a surrogate marker for tissue vascularization [2,3].

Despite these advancements, the characterization of physiological pulmonary iodine distribution remains underexplored, with most research primarily focusing on pathological conditions and iodine distribution in organs. IMs are widely used to evaluate pulmonary perfusion, supporting the detection of acute pulmonary embolism (PE) [4] and additionally enhancing the differentiation between acute PE and chronic thromboembolic pulmonary hypertension through quantification of pulmonary perfusion patterns [5,6]. Similarly, DECT and iodine quantification have facilitated the workup of pulmonary masses [7,8] and improved the detection of pleural carcinomatosis [9]. Lung perfusion analysis via DECT is instrumental in understanding pathophysiological pulmonary transformations in lung conditions like pneumonia and atelectasis [10,11]. For instance, in COVID-19 pneumonia, IMs reveal an extremely heterogeneous pattern, potentially reflecting inhomogeneous inflammatory processes [12].

Previous studies involving organs other than the lung have validated DECT-derived iodine quantification as reliable for assessing organ perfusion. However, they mostly highlight the need for normalization due to variations in contrast medium doses and suggest considering patient age and sex when interpreting iodine concentration cutoffs [13,14,15].

Several methods exist for quantifying pulmonary perfusion, each with advantages and limitations. Ventilation/perfusion (V/Q)-scintigraphy with SPECT is still considered the current gold standard in assessing pulmonary perfusion, combining injection and inhalation of a radioactive tracer and offering accurate three-dimensional imaging of pulmonary perfusion, but is limited in spatial and temporal resolution [16]. MRI offers high-resolution images and the advantage of not using ionizing radiation, but it is less commonly used due to high costs and its limited availability [11,17]. Recent advances in DECT, through the generation of IMs, enable the detection of pulmonary perfusion defects with good correlation to SPECT with V/Q-scintigraphy [18,19]. Despite the promising applications, the quantitative interpretations of IMs are frequently hindered by factors such as intra-patient variability with a lack of standardized acquisition and normalization protocols, limiting its clinical application [20,21].

Despite the evident benefits in research and possible clinical application, a detailed characterization of physiological iodine distribution within the healthy lung is lacking. This study aims to address this gap by automating pulmonary iodine quantification in a lung-healthy cohort, thereby assessing demographic effects on pulmonary iodine distribution.

## 2. Materials and Methods

### 2.1. Patient Enrollment

This single-center, retrospective cohort study was conducted in accordance with the Declaration of Helsinki and local institutional review board (Ethics Committee of the University of Cologne), and the need for informed consent was waived. For this single-center, retrospective study the patient collective included patients ≥ 18 years, who received portal venous phase staging CT scans due to a clinical indication with a dermato-oncological disease, but without any current tumor burden. The patients received at least two CT scans in the period from 2016 to 2019. All scans were checked for potential lung diseases and parenchymal abnormalities, and those with findings were excluded.

### 2.2. Image Acquisition

All patients were scanned in supine position on a commercially available dual-layer dual-energy CT (dlDECT) (IQon, Philips Healthcare, Best, The Netherlands). A total of 100 mL of iodinated contrast media (Accupaque, 350 mg/mL; GE Healthcare, Chicago, IL, USA) was injected via a 20-G catheter in the antecubital fossa. Bolus tracking in the descending thoracic aorta with a threshold of 150 HU and a delay of 50 s for the portal venous phase were applied. Images were acquired using the following settings: tube voltage 120 kV; tube current modulation (DoseRight 3D-DOM, Philips Healthcare, Best, The Netherlands), rotation time 0.33 s, craniocaudal scan direction, collimation 64 mm × 0.625 mm, pitch 0.671.

### 2.3. Image Reconstruction and Postprocessing

Conventional images (CIs) were reconstructed using the standard lung preset (IDose^4^, denoising level 3/7, kernel YA, Philips Healthcare, Best, The Netherlands). Iodine maps (IMs) were processed from spectral-based data using a hybrid-iterative spectral reconstruction method (spectral, denoising level 3/7, filter B, Philips Healthcare, Best, The Netherlands). For all datasets, slice thickness was set to 2 mm and section increment to 1 mm. All images were reconstructed with a matrix of 512 × 512.

### 2.4. Data Acquisition

CIs were used for assessing the volumetry of the lung by certified software (IntelliSpace Portal, Version 11.0, COPD, Philips Healthcare, Best, The Netherlands). Segmentation results were manually confirmed by a radiologist with two years of experience in chest computed tomography (T.S.). The volumetry of each lung was merged with CIs and IMs using a dedicated research platform (IntelliSpace Discovery, Version 3.0, Philips Healthcare). To avoid large pulmonary vessels, a thresholding step was performed using the iodine concentration in the left atrium before assessing pulmonary iodine concentration following a workflow published previously [22].

Volume and mean iodine concentration were calculated for each lung lobe for every examination. Additionally, for each examination a circular ROI was placed in the portal vein proximal to the bifurcation and main pulmonary artery (mPA), and mean iodine concentration was exported as well. The mPA was selected because it represents the direct upstream vessel supplying the pulmonary circulation. In contrast, the portal vein was included as a stable intra-abdominal reference vessel, though its hemodynamic relationship with the lungs is indirect.

**Figure 1:**
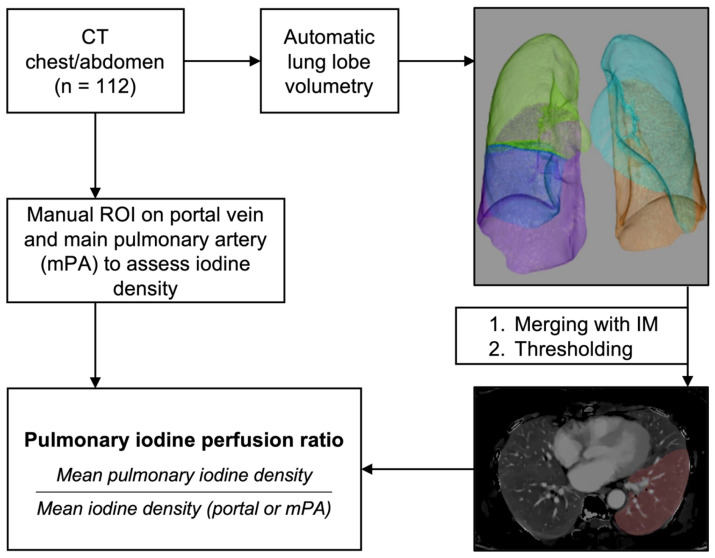
Automated volumetric assessment of lung lobes and lobe-specific iodine density. Top right: Exemplary automated lobar segmentation with color-coded volumetric masks: right upper lobe (green), right middle lobe (dark blue), right lower lobe (purple), left upper lobe (light blue), left lower lobe (orange). Bottom right: Volumetric mask of the left lower lobe superimposed on the iodine map. The vascular structures are excluded via a thresholding process.

Pulmonary iodine perfusion ratios (PIPRs) were calculated using mean pulmonary iodine density and the iodine density of portal vein or mPA (Figure 1).

$$Pulmonary\;iodine\;perfusion\;ratio\;(PIPR)=\frac{Mean\;pulmonal\;iodine\;density\;in\;mg/mL}{Mean\;iodine\;density\left(portal\;or\;mPA\right)\;in\;mg/mL}$$

Lung volumes of a total of 112 patients were automatically assessed using a commercially available tool with individual volumes for each lung lobe. The volumetric file was then merged with a dual-layer DECT-derived iodine map. Pulmonary vessels were excluded through thresholding against iodine concentration in the left atrium. Additionally, iodine concentration was assessed in portal vein and main pulmonary artery (mPA) to calculate the specific pulmonary iodine perfusion ratio.

### 2.5. Statistical Analysis

Statistical analysis and graphical visualization were performed using JMP (v17.2.0, SAS Institute, Cary, NC, USA) and GraphPad Prism (v10.2.0, GraphPad Software, Boston, MA, USA) as well as R (v4.3.0, R Foundation for Statistical Computing) [23] with RStudio (2023.3.0.386, Posit Software, PBC, Boston, MA, USA). Statistical significance was defined as *p* < 0.05.

Data was tested for normal distribution using Shapiro–Wilk. Parametric data was compared with the Student’s *t*-test whereas the Mann–Whitney-U test or Wilcoxon signed rank test was utilized for nonparametric unpaired or paired data respectively. The Friedman test was used for nonparametric comparisons of multiple paired data. To check for potential correlations, the Pearson coefficient was used for parametric data and the Spearman coefficient for nonparametric data. Bivariate regression was performed to evaluate dependencies.

## 3. Results

### 3.1. Patient Demographics

Mean patient age was 60.3 years (±16.6 years) with 30 patients < 50 years, 44 patients between 50 and 70 years, and 38 patients > 70 years. In total, 53% of the patients were female. Average time between the two CT scans was 262.0 days (±169.2 days). The mean lung volume was 4502.6 cm^3^ (±1294.5 cm^3^), with female patients having a mean lung volume of 3896.5 cm^3^ (±928.0 cm^3^) compared to 5177.3 cm^3^ (±1317.6 cm^3^) in males (Table 1).

### 3.2. Value of Normalization of Pulmonary Iodine Distribution

The quantification of total iodine content in the lung demonstrated substantial variability (mean: 3347.9 mg ± 596.6 mg), primarily influenced by individual variations in lung volumes. Therefore, the study focused on the relative iodine density. Given the administration of a fixed volume of iodine-containing contrast medium independent of total body weight and body mass index, it was necessary to normalize the relative iodine density to account for this variation. This normalization process involved adjusting the total pulmonary iodine levels relative to either the iodine concentration in the portal vein or the mPA. This step is commonly recommended in previously published works dealing with iodine density in varying organ systems [13,14].

**Figure 2:**
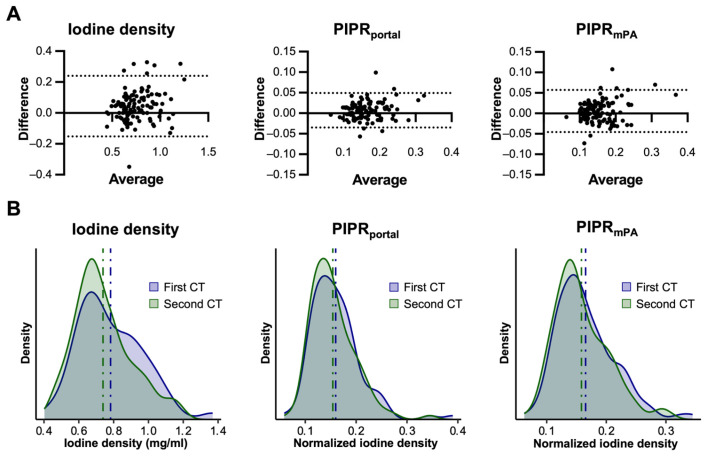
Normalization of lung iodine density for portal iodine concentration enhances reproducibility. (**A**) Bland–Altman plots illustrating the differences between the two DECT scans within patients for iodine density (r = 0.82) and normalized pulmonary iodine perfusion ratios (PIPRs). Normalization was performed against iodine density of the portal vein (PIPR_port_, r = 0.81) and the main pulmonary artery (PIPR_mPA_, r = 0.87). The dotted lines indicate the 95% limits of agreement (mean difference ± 1.96 SD). (**B**) Histograms showing the distribution of iodine density before and after normalization against the iodine density of the portal vein (PIPR_portal_) and mPA (PIPR_mPA_). The alignment of the distribution improved following normalization against portal vein and main pulmonary artery iodine levels. The dashed lines represent the mean values.

Prior to normalization, the correlation coefficient (*r*) of the mean iodine concentration between the two CT scans was 0.82. Further analysis demonstrated that assessing the pulmonary perfusion ratio through normalization using the iodine concentration in the mPA (pulmonary iodine perfusion ratio, PIPR_mPA_) improved the correlation coefficient *r* to 0.88, while normalization against the portal iodine level did not alter the correlation (*r* = 0.81) (Figure 2A,B). As a result, subsequent analyses were conducted using the PIPR_mPA_ normalization method to ensure accuracy and reliability in the assessment of pulmonary iodine distribution.

### 3.3. Demographic and Physiological Variations in Pulmonary Iodine Perfusion Ratios

**Figure 3:**
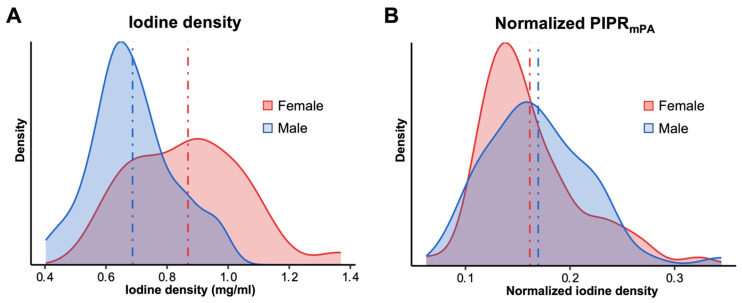
Influence of normalizing pulmonary iodine density on sex effect. Histogram plot of the iodine density distribution between males and females before (**A**) and after (**B**) normalization against the iodine concentration of the main pulmonary artery (mPA) revealed a significantly higher iodine level in females (0.87 mg/mL ± 0.18 mg/mL) compared to males (0.69 mg/mL ± 0.13 mg/mL, *p* < 0.001). Normalization against mPA iodine level effectively reduces and even slightly reverses this difference with no significant difference remaining (*p* = 0.28). The dashed lines represent the mean values.

Females demonstrated smaller mean lung volumes (3.9 L ± 0.9 L) compared to males (5.2 L ± 1.3 L) and exhibited higher mean iodine density (0.87 ± 0.13 mg/mL vs. 0.69 ± 0.18 mg/mL, *p* < 0.001). However, after normalizing the total iodine density against the iodine level in the mPA, these differences were effectively neutralized, yielding an adjusted PIPR_mPA_ of 0.16 (±0.048) for females and 0.17 (±0.05) for males (*p* = 0.28; Table 1 and Figure 3).

**Figure 4:**
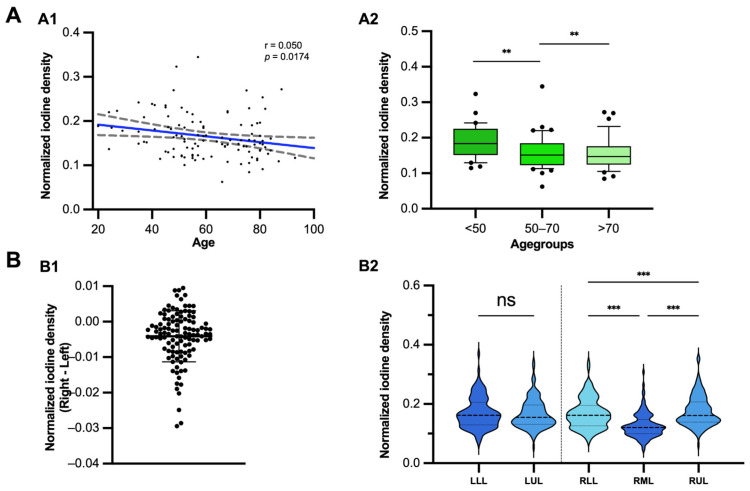
Normalized lung iodine density demonstrates expected physiological variations with age and anatomical distribution. (**A**) Regression model of the normalized iodine density against biological patients age (**A1**). Pulmonary iodine perfusion ratios demonstrated a significant decline with increasing age (*p* < 0.05). Box plots of normalized iodine density and different age groups (**A2**). Notably, younger age groups (<50 years) exhibited significantly higher perfusion ratios compared to older patients. (**B**) Scatter dot plot of interpatient differences of the normalized pulmonary iodine perfusion ratio (PIPRmPA) between the right and the left lung (Right minus Left) (**B1**) and violine plots of PIPRmPA for each individual lung lobe (**B2**). Slightly higher values for the left lung (**B1**) compared to the right lung. Additionally, in the supine position, the more upward right middle lobe is less perfused compared to the lower and upper lobe. No significant difference was found between the left upper and lower lobe. Statistical significance is indicated as follows: ** *p* < 0.01, *** *p* < 0.001, ns = not significant.

A decline in the pulmonary perfusion ratio was also evident with increasing age, with a significant difference observed between individuals younger than 50 years (0.189 ± 0.048) and those predominantly older than 70 years (0.156 ± 0.046, *p* < 0.01) (Figure 4A).

Regarding regional distribution a small, yet significant (*p* < 0.001) difference was observed in the perfusion ratio between the left lung (0.163 ± 0.05) and the right lung (0.159 ± 0.05). When examining differences across lung lobes, the right middle lobe (RML) consistently showed the lowest pulmonary perfusion ratio (0.127 ± 0.04). In contrast, the other lobes exhibited relatively similar perfusion ratios, with the highest observed in the right upper lobe (0.181 ± 0.08) and slightly lower ratios in the left upper lobe (0.166 ± 0.05) compared to the left lower lobe (0.170 ± 0.05) (Figure 4B).

## 4. Discussion

To our knowledge, this is the first study to evaluate iodine density in healthy lung parenchyma within a cohort of patients without tumor or lung abnormalities. This study has important implications, particularly when considering the vast potential for diagnosing and monitoring a spectrum of pulmonary conditions, ranging from malignancies, chronic obstructive pulmonary disease (COPD), to inflammatory diseases such as COVID-19 pneumonia, as well as pulmonary circulatory disorders including pulmonary embolism and pulmonary hypertension. One major aspect of our methodology was the normalization of iodine measurements to the concentration found in the mPA, which effectively minimized the variability inherent in iodine distribution, a critical step in enhancing the precision and utility of these measurements in clinical practice.

One of the advantages of dlDECT is its ability to accurately quantify iodine levels in tissue [1]. This capability enables the opportunity to get a snapshot of organ perfusion, including the perfusion of lung parenchyma and pulmonary lesions. The utility of pulmonary perfusion analysis has been validated in various studies, especially against the reference standard of V/Q-scintigraphy [18]. In recent years, this snapshot perfusion has proven to enhance the diagnostic accuracy for acute pulmonary embolism (PE) [24]. Iodine maps also aid in distinguishing between acute PE and chronic thromboembolic pulmonary hypertension [5]. Additionally, this snapshot perfusion may serve as a supplementary tool in oncological imaging. The move towards quantification is driven by the need to overcome the limitations of conventional imaging biomarkers, which often fail to capture the dynamic nature of tumor physiology and response to therapy. Chae et al. demonstrated that DECT analyses of iodine content can aid with differentiating between benign and malignant lung lesions [25]. Furthermore, Iwano et al. demonstrated that iodine concentration in primary lung cancer is inversely proportional to cellular differentiation, with highly differentiated tumors exhibiting lower iodine levels, likely due to increased tumor necrosis [26].

Despite all these various use cases for pulmonary DECT-derived snapshot perfusion, little is known about the physiological distribution of iodine in the lungs. Our data indicate that normalizing iodine concentration relative to blood vessel levels is indispensable for accurate assessment of pulmonary perfusion in the portal venous phase. While normalization strategies have been validated in previous studies for abdominal organs and bone marrow, their application to pulmonary imaging has not yet been established [13,14]. Iodine concentration measurements are known to exhibit substantial intra-patient variability, which can be partially mitigated by current normalization techniques [27]. When evaluating various blood vessels for this purpose, our findings show that normalization against the iodine level in the mPA demonstrates greater reliability compared to the portal vein. The differences between normalization against the iodine concentration in the mPA and portal vein in the portal venous phase may be attributed to the fact that the mPA is directly upstream of the lung and thereby provides a more relevant reference point. Normalization likely compensates for variations in body weight and size, considerations that are critical given the administration of a fixed volume of iodine-containing contrast medium in clinical practice. The necessity of normalization is further evidenced by its capacity to reduce sex effects within our dataset, which otherwise appear to be driven by the aforementioned variations. Nonetheless, our findings also demonstrate that pulmonary iodine perfusion ratios (PIPRs) progressively decline with increasing age, which may reflect the established effect of increasing ventilation–perfusion inequality with increasing age [28]. Interestingly, we also observed that the PIPR from portal venous phase CT scans was lowest in the RML compared to all other lung lobes. Given that the patients were scanned in a supine position, this may reflect the well-known gravity dorsal-to-ventral gradient. This phenomenon, where perfusion in the supine position is typically greater in the dorsal regions of the lung, has been documented in several studies, including those focused on the physiological distribution of pulmonary blood flow and the interpretation of V/Q scans as well as for IMs in DECT scans of the pulmonary arteries, suggesting a consistent pattern across different phases of contrast medium application [5,29,30,31]. As stated above, IMs derived from DECT scans during dedicated CTPA enhance the detection of pulmonary artery embolisms, e.g., by visualizing the resulting perfusion deficit [4]. In addition to symptomatic, acute pulmonary artery embolisms, there are also incidental pulmonary artery embolisms that are detected during CT staging examinations. A study conducted in a large hospital in Germany showed that approximately 2.7% of oncological patients undergoing follow-up staging examinations had an incidental, asymptomatic pulmonary artery embolism, which could have direct treatment implications [32]. Given this, it would be valuable to investigate whether the additional utility of IMs could increase the detection of incidental pulmonary embolisms in CT staging examinations as well, which are mostly performed in the portal venous phase. Our study provides a groundwork for such an evaluation by providing reference values of a cohort of lung-healthy individuals.

Our research has several limitations in addition to the retrospective study design. First, the inclusion of patients referred for thoracoabdominal staging CT, specifically for dermato-oncological reasons, introduces a selection bias; however, the CT scans were checked by an experienced radiologist for any tumor burden as well as for any suspicious changes in lung parenchyma. Still, no correction for any cardiovascular diseases, e.g., heart failure, has been performed, which could influence the iodine distribution as well. Second, the focus on portal venous phase examinations, while necessary for the scope of our study, underscores the need for a broader application of imaging protocols such as pulmonary angiograms as well as arterial phases and the comparison between these phases. Furthermore, our analysis was performed in a single center at one specific CT device only, which on the one hand increases the reproducibility of our results. On the other hand, however, it remains unclear whether the results can be applied to other sites.

In conclusion, by looking at the pulmonary iodine concentration in a lung-healthy cohort in the portal venous phase, this is the first study providing quantitative reference values for iodine levels and evaluating normalization techniques. Thereby it can serve as a tool for future research and clinical application of DECT in pulmonary imaging. Additionally, it highlights the effect of demographics and sex on pulmonary perfusion. Nonetheless, further studies are necessary to evaluate PIPRs with DECT on different devices and for different pathologies, for which the values published here might serve as a benchmark.

## Figures and Tables

**Table 1:** Effects of demographics and physiology on pulmonary perfusion.

		Lung Volume (cm^3^)		Mean mPA Iodine Concentration (mg/mL)		Mean Pulmonary Iodine Concentration (mg/mL)		Normalized PIPRmPA		*p*-Values PIPRmPA		
	N	Mean	SD	Mean	SD	Mean	SD	Mean	SD			
Total	112	4502.6	1294.5	4.95	1.28	0.78	0.19	0.17	0.05			
female	59	3896.5	928.0	5.61	1.24	0.87	0.18	0.16	0.05	f vs. m		0.28
male	53	5177.3	1317.6	4.23	0.87	0.69	0.13	0.17	0.05			
Age group												
<50	30	4243.7	1254.4	4.64	1.25	0.84	0.20	0.19	0.05	<50 vs. 50–70		<0.01
50–70	44	4850.0	1342.8	4.90	1.22	0.74	0.16	0.16	0.05	50–70 vs. >70		0.74
>70	38	4304.7	1208.6	5.27	1.33	0.79	0.19	0.16	0.05	<50 vs. >70		<0.01
Left	112	2060.4	627.3			0.79	0.19	0.17	0.05	Left vs. Right	<0.001	
Right		2442.2	684.5			0.78	0.18	0.16	0.05			

Data is given as mean with standard deviation (SD). Pulmonary iodine perfusion ratio normalized against the iodine concentration in the main pulmonary artery (PIPR_mPA_).

## Data Availability

The raw data supporting the conclusions of this article will be made available by the authors on request.

## Abbreviations

The following abbreviations are used in this manuscript:

DECT	Dual-energy computed tomography
PIPRs	Pulmonary iodine perfusion ratios
mPA	Main pulmonary artery
dlDECT	Dual-layer dual-energy CT
PE	Pulmonary embolism
SPECT	Single-photon emission computed tomography
IMs	Iodine maps
